# Bipolar Disorder in Pregnancy: A Challenging Case Managed with Maintenance Electroconvulsive Therapy

**DOI:** 10.1192/j.eurpsy.2024.274

**Published:** 2024-08-27

**Authors:** B. Arribas-Simon, M. J. Mateos-Sexmero, O. Martin-Santiago, P. Andres-Olivera, B. Rodriguez-Rodriguez, P. Martinez-Gimeno, N. Navarro-Barriga, T. Jimenez-Aparicio, P. Fernandez-Pando, M. Andreo-Vidal

**Affiliations:** ^1^Psychiatry, Hospital Clinico Universitario, Valladolid; ^2^Psychiatry, Complejo Asistencial Universitario, Salamanca; ^3^Hospital Clinico Universitario, Valladolid, Spain

## Abstract

**Introduction:**

Pregnancy is a high-risk period for major affective disorders and can lead to a destabilizing period for our patients. Standard pharmacological strategies must be carefully evaluated due to potential teratogenic or side effects. We present a case of bipolar disorder type I with challenging-to-control maniac episodes during pregnancy, which has required Electroconvulsive Therapy for its management.

**Objectives:**

Presenting maintenance electroconvulsive therapy (ECT) as a safe and effective therapeutic strategy during pregnancy, with the presentation of a case in which it has been administered every 3 weeks from the second trimester until the baby’s birth at 37 weeks

**Methods:**

This concerns a 28-year-old immigrant woman, married, with a 10-year-old child. She was diagnosed with bipolar disorder type I at the age of 16 when she experienced her first manic episode in her country of origin. Subsequently, during her first pregnancy, she required hospitalization for electroconvulsive therapy (ECT) treatment, with a positive response after a single session. She remained stable for several years without maintenance pharmacological treatment or follow-up until the ninth week of her second pregnancy when she experienced a manic episode requiring hospitalization.

**Results:**

She was initially treated with Olanzapine and Lorazepam with a positive response, but three weeks later, she was readmitted with a similar episode. These decompensations occurred almost monthly, leading to the consideration of introducing mood stabilizers after the first trimester. However, due to the patient’s severe hyperemesis gravidarum, this stabilizing treatment was ruled out due to the difficulty in controlling its blood levels and the associated risk of intoxication. During the fifth admission at the 20th week of gestation, the decision was made to initiate ECT treatment, which yielded an excellent response and subsequent maintenance.

**Conclusions:**

The indications for electroconvulsive therapy (ECT) during pregnancy are the same as in the rest of adult patients. In individuals with a psychiatric history, it is possible for a relapse of mental illness to occur during pregnancy, although the risk is considerably higher during the postpartum period. ECT is considered an effective and safe treatment option in all three trimesters of pregnancy and the postpartum period. During the informed consent process, patients should be informed about the potential impact of ECT as well as alternative treatment options.

**Disclosure of Interest:**

None Declared

